# Imaging of the Heart: Potential Application to Alcohol-Induced Heart Disease

**Published:** 1995

**Authors:** Steven R. Bergmann

**Affiliations:** Steven R. Bergmann, M.D., Ph.D., is a professor of medicine and radiology based at the Cardiovascular Division, Washington University School of Medicine, St. Louis, Missouri

**Keywords:** diagnostic imaging, heart function, muscle, metabolism, fatty acids, alcoholic cardiomyopathy, myocardium, single photon emission computed tomography, positron emission tomography, magnetic resonance imaging

## Abstract

In addition to its effects on the brain and other organs, chronic heavy alcohol use can lead to cardiovascular diseases including hypertension. Another consequence of heavy drinking is alcohol-induced heart muscle disease, which leads to a diminished pump function of the heart. Several mechanisms related to alcohol and its metabolism in the body can contribute to alcohol-induced heart muscle disease by interfering with the heart’s metabolism and energy production and thereby impairing heart muscle function. Noninvasive imaging techniques can help researchers and clinicians to monitor alcohol’s effects on the heart and its metabolism. For example, one study found that increasing amounts of alcohol reduced the uptake of fatty acids—the primary fuel used by the heart muscle—into the heart. Other investigators have demonstrated that the heart’s metabolism of fatty acids is impaired in patients with alcohol-induced heart muscle disease. Although the potential of imaging techniques for studying alcohol-induced heart muscle disease has not yet been fully explored, these approaches are likely to play an increasingly important role in the future.

Although liver disease is by far the most prevalent physiological consequence of alcohol abuse and dependence, 1 percent to 2 percent of all chronic alcohol abusers also develop overt heart disease, such as a thickened heart resulting from alcohol-induced high blood pressure or heart failure (i.e., the heart’s inability to pump sufficient blood to meet the body’s needs) (Bing 1982; Lieber 1995; Preedy and Richardson 1994; Urbano-Marquez et al. 1989). Alcohol-induced heart disease afflicts more than 200,000 Americans and accounts for approximately 3 percent of all hospital admissions for heart problems in the United States (Bing 1982; Lieber 1995; Preedy and Richardson 1994). Even alcohol-abusing patients without overt signs of heart failure often may experience signs of depressed cardiac function during increased activity (e.g., shortness of breath when climbing stairs).

Alcohol can have many effects on the heart and the cardiovascular system: It can depress the heart’s pumping ability, induce irregular heartbeats, increase blood pressure, and alter the fat content and metabolism of the heart muscle. The exact mechanism(s) by which alcohol induces these effects, however, remains unclear. This lack of knowledge is due, in part, to the scarcity of available research methods that allow researchers to explore the heart’s structure and function without surgery (i.e., noninvasively). New, noninvasive imaging techniques developed during the past decade promise to be immensely important in evaluating changes in the heart’s blood flow and metabolism that are induced by alcohol consumption and other conditions.

Although imaging techniques have not yet been applied extensively to the study of alcohol-induced diseases of the heart and other organs, in the future this technology should provide insight into factors that underlie alcohol-induced disease. These techniques also may help elucidate how alcohol-induced changes in heart function respond to abstinence and other therapeutic interventions. This article examines the relationship between alcohol and heart disease and reviews some of the structural and functional imaging techniques being used to study disorders of the heart and cardiovascular system. The results of studies using imaging technology to investigate the abnormalities underlying cardiac dysfunction in alcoholics also are presented.

## The Relationship Between Alcohol and Heart Disease

Many studies have demonstrated that modest alcohol consumption (i.e., one to two drinks^1^ per day) may help protect the heart from coronary artery^2^ disease by altering the levels of lipoproteins^3^ in the blood, enhancing the dissolution of dangerous blood clots, and reducing the aggregation of certain blood cells (Bing 1982; Lieber 1995; Preedy and Richardson 1994). In contrast, heavy drinking (i.e., five to six drinks per day) can result in cardiomyopathy and a decrease in the heart’s pumping function. The term “cardiomyopathy” refers to any disease of the heart muscle (i.e., myocardium), independent of its cause. In alcoholics, this condition is commonly called alcohol-induced heart muscle disease, or alcoholic cardiomyopathy. Because alcohol-induced heart muscle disease does not differ physiologically from other kinds of cardio-myopathy, its diagnosis usually is based on a history of alcohol abuse coupled with the exclusion of other risk factors.

Cardiomyopathy primarily is characterized by an enlargement of the heart. This enlargement usually appears as a thickening (i.e., hypertrophy) or—in advanced stages—as a thinning (i.e., dilation) of the myocardium and especially of the left ventricle, the chamber that pumps blood received from the lungs to the rest of the body (for a review, see Rubin and Doria 1990).

Hypertrophy usually occurs because alcohol-induced increases in blood pressure force the left ventricle to work harder to pump blood. Dilation generally is caused by alcohol-induced changes in the metabolism of the heart muscle cells. Such changes reduce the cells’ ability to contract and, thus, expel blood from the ventricle. (Alcohol’s effects on heart-cell metabolism will be described in more detail below.) Consequently, after each contraction, more blood than normal remains in the ventricle, combining with the blood entering the ventricle from the lungs.

The increased blood volume in the ventricle, combined with other reactions in the body to counteract the reduced blood flow from the heart, eventually leads to the enlargement of the left ventricle, as well as the other chambers of the heart, and to an increase in heart muscle mass. However, because the coronary arteries cannot supply enough blood to sustain the heart’s increased demand for oxygen and other nutrients, the enlarged heart cannot pump sufficient blood to the body. The patient eventually becomes weaker and even bed-ridden. Thus, alcohol-induced metabolic changes result in structural changes in the heart muscle with potentially serious consequences.

In addition to the long-term effects of heavy alcohol consumption, acute alcohol intoxication can induce disturbances in the heart rhythm, which in turn can decrease the effectiveness of the heart’s pumping capacity. In rare instances, severe heart rhythm disturbances can result in sudden death. Patients with non-insulin-dependent (type II) diabetes may be especially susceptible to the toxic effects of alcohol. After drinking, these patients exhibit increased levels of alcohol and its degradation product, acetaldehyde (see below), in their blood. They also have a higher mortality and morbidity from alcohol-induced disease (Balkau et al. 1991, 1992; Bing 1982; Lieber 1995; Preedy and Richardson 1994). For example, in patients with diabetes the relative risk of death related to alcoholism and cirrhosis was 13 times that of nondiabetics and correlated with the level of alcohol consumption. Moreover, for unknown reasons, women generally appear to be more susceptible to alcohol’s effects on the heart than men (Urbano-Marquez et al. 1995).

Changes in the myocardium associated with early stages of alcohol-induced cardio-myopathy usually are reversible, whereas in late disease stages the changes typically are irreversible. For example, if a person who has developed heart muscle hypertrophy without dilation stops drinking, his or her myocardium can return to its normal size and pumping capacity. Once dilation occurs, however, it usually is irreversible, even with abstinence. Consequently, non-invasive techniques that could detect specific signs of alcohol-induced heart muscle disease early not only would facilitate diagnosis but also would allow clinicians to monitor disease progression or regression in response to therapy. In addition, the results of such analyses would provide insight into the development of disease and help determine why some chronic alcohol abusers are more susceptible to cardiac disease. Finally, characterization of the alcohol-induced changes in the body’s blood flow and biochemistry should help distinguish, from a diagnostic, pathogenic point of view, between alcohol-induced heart muscle disease and other forms of cardiomyopathy. Such information will give clinicians the tools they need to treat the specific metabolic abnormalities which lead to the different kinds of cardiomyopathy. Noninvasive imaging techniques should allow researchers and clinicians to answer many of these questions.

## Detection of Structural and Functional Changes in the Heart

Several imaging techniques can be used to define the heart’s structure and monitor its pumping ability, including echocardiography, radionuclide ventriculography (RVG), and magnetic resonance imaging (MRI). During echocardiography, ultra-high-frequency sound waves are transmitted to the heart and reflected back to a receiver. For RVG, red blood cells are labeled with a small amount of a radioactive tracer; special cameras then measure the radioactivity and its distribution in the blood to assess the heart’s contracting ability. MRI is based on the observation that certain chemical elements (e.g., hydrogen) emit an electromagnetic signal when placed within a magnetic field and excited by radio waves. This signal then can be converted into an image. (For more information on MRI, see the article by Doria, pp. 261–265). Because MRI generally relies on signals emitted by hydrogen, which is an extremely abundant element in the body, it produces exquisite anatomical pictures, which have been used to define the heart’s structure and function. The drawback of MRI, however, is that it is much more expensive than echocardiography or RVG.

Echocardiography, RVG, and MRI all are useful in demonstrating reductions in the heart’s ability to pump blood; additionally, each of these techniques helps to define the heart’s anatomic structure (e.g., distinguish hypertrophy from dilation). They can measure the thickness of the heart muscle, the size of the ventricles, and the amount of blood in the heart that is pumped outward during the heart’s contraction (i.e., the ejection fraction, a useful measure of the heart’s functioning). In addition, these techniques can monitor changes in heart structure and function that occur over time. These analyses usually are performed when a patient has complaints, such as diminished exercise ability or increasing shortness of breath after exertion.

Although these techniques allow physicians to diagnose cardiomyopathy and monitor the effects of treatment, they cannot determine the biochemical disorders underlying impaired contraction. This information can be obtained, however, from functional imaging technologies, such as single-photon imaging techniques, positron emission tomography (PET), or more advanced MRI approaches. Each of these approaches has particular advantages and limitations (table 1).

## Detection of Metabolic Changes in the Heart

To understand the basis of imaging techniques that monitor the metabolism of a tissue, a brief review of the heart’s metabolism is necessary.

### Normal Myocardial Metabolism

To maintain constant, rhythmic contractions, the heart must receive energy from vital nutrients. The heart normally uses a type of fat molecules called fatty acids to provide most of these energy needs. A second major energy source is glucose, a sugar. For example, after a meal, glucose levels in the blood increase, which, in turn, suppresses the release of fatty acids from the body’s peripheral tissues. As a result, the heart’s use of fatty acids decreases while its use of glucose increases. In addition to fatty acids and glucose, the heart can use a wide range of other substances as energy sources, including acetate, which is a breakdown product of alcohol (see below). Because only small amounts of these alternative substances usually are present in the blood, however, the heart uses them less frequently than fatty acids or glucose.

In a healthy person, the level of blood flow and metabolism in the myocardium is closely linked to the amount of work performed by the heart. For example, when a runner is in a race, the heart must pump more blood to provide energy for the skeletal muscles. The blood flow to the heart muscle also increases to two to three times the normal rate to deliver additional fuel needed by the heart. This allows the heart to increase its pumping activity and thus to meet the body’s increased energy needs. The biochemical processes that provide energy for the contraction of the myocardium also are accelerated.

Several disease processes can interfere with the blood flow to the heart or disrupt the heart’s ability to metabolize fuel to energy. In Western societies, for example, deposits of fat droplets and other substances on the walls of the coronary arteries (i.e., atherosclerotic coronary artery disease) is quite prevalent and leads to the narrowing of these arteries. As coronary atherosclerosis progresses, the blood flow to the heart may be normal under resting conditions. However, when the body’s energy needs increase (e.g., when climbing stairs or carrying groceries), the narrow arteries restrict blood flow to the heart and thus limit the delivery of fuel. The heart cannot meet the increased metabolic needs, and the patient experiences chest pain (i.e., angina pectoris), which generally causes the patient to cease the increased activity by either sitting or lying down. This decreases the amount of work performed by the heart, and blood flow to the heart returns to normal levels.

When a patient has a heart attack (i.e., myocardial infarction), blood flow through a coronary artery is completely blocked, usually by a blood clot in conjunction with atherosclerotic narrowing. This blockage prevents the blood from delivering needed nutrients and oxygen to the myocardium. As a result, the myocardium cannot produce enough energy to sustain heart contraction; effective contraction ceases. Moreover, the affected part of the heart muscle dies from a lack of oxygen unless the condition is treated immediately.

### Alcohol’s Effects on Myocardial Metabolism

**Figure 1:**
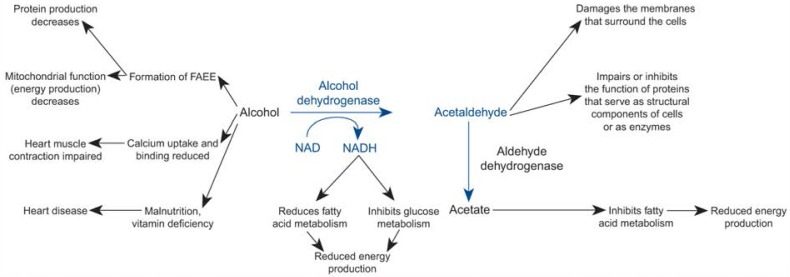
Schematic diagram of important metabolic effects of alcohol and its degradation products, acetaldehyde and acetate. Many of these changes affect the metabolism of fatty acids, the main fuel for the heart muscle’s energy production. Fatty acid ethyl esters (FAEE) are potentially toxic derivatives of fatty acids. Nicotinamide adenine dinucleotide (NAD) is a co-enzyme that plays an accessory role in enzyme catalysis. NADH is the reduced form of NAD.

Ingestion of alcohol induces numerous biochemical reactions in the body. Alcohol is rapidly absorbed from the gastrointestinal system and distributed throughout the body. Nearly all of the alcohol consumed is metabolized in the liver by two enzymes, alcohol dehydrogenase (ADH) and aldehyde dehydrogenase (ALDH) (figure 1). ADH converts alcohol into acetaldehyde, which in turn is metabolized by ALDH to form acetate, a fatty acid containing two carbon atoms. Acetate can be used as an energy source by most tissues in the body, including the myocardium. When blood alcohol levels are high, however, another group of enzymes also begin to convert alcohol into acetaldehyde and water, leading to the accumulation of acetaldehyde in the body.

Alcohol, its degradation products, and other substances produced during alcohol metabolism all can have adverse effects on the body’s tissues, including the myocardium. These effects include (see also figure 1):

- Alcohol can combine with fatty acids to form so-called fatty acid ethyl esters (FAEE) (Lange et al. 1981). FAEE can form in all tissues, including the myocardium, and inhibit the function of cellular components (i.e., the mitochondria) that are necessary for cell energy production. In addition, FAEE decrease the production of proteins essential for cell structure and function. Alcohol also may interfere with the myocardial uptake and binding of calcium, a process that is important for muscle contraction.
- Acetaldehyde has several direct toxic effects on the body. For example, acetaldehyde inhibits the repair of proteins that form important structural components of the cells. It also induces damage to the membranes surrounding the cells (Bing 1982; Rubin 1993; Preedy and Richardson 1994; Lieber 1995).
- Acetate levels in the blood, which normally are relatively low, can increase after alcohol consumption. Although acetate can serve as an energy source for myocardial metabolism, high acetate levels ultimately can impair the heart’s ability to use normal fuels, such as fatty acids, for metabolism.
- Other substances that are produced during the degradation of alcohol can interfere with the cells’ normal metabolism of fatty acids and glucose and thereby reduce energy production.

Clearly, ingestion of alcohol has multiple, wide-ranging effects that can alter the heart’s metabolism and contraction ability both directly and indirectly. Moreover, alcoholics often have nutritional deficiencies, including vitamin deficiencies, that also may lead to heart disease.

The influence of the individual alcohol effects on the development of alcohol-induced heart disease, the correlation between the amount of alcohol consumed and the severity of these effects, and the ability of abstinence or other therapeutic interventions to reverse alcohol-induced changes are still unknown. Metabolic imaging techniques, such as single-photon imaging and PET, should be extremely helpful in addressing these issues.

### Single-Photon Imaging

For analyses of heart function using single-photon imaging techniques, a small, harmless amount of a radiolabeled^4^ substance (i.e., a radioisotope), typically a radiolabeled fatty acid, is injected into the patient’s blood. The radioisotopes used for these techniques are not normally found in the body and, therefore, are incorporated into compounds that are similar, but not identical, to physiological substances. The heart can use the radiolabeled substances (i.e., tracers), almost the same way as the normal compounds. Using a special camera to record the radiation emitted from the heart and convert the information into an image, the clinician or researcher can analyze the heart’s uptake and metabolism of the tracer. Depending on the device used to record the radiation, the imaging techniques are referred to as single photon scintigraphy and single-photon emission computed tomography (SPECT). (For more information on SPECT, see the article by Doria, pp. 261–265.)

### Single-Photon Imaging To Study Alcohol’s Effects

To study fatty acid metabolism using single-photon techniques, clinicians have employed fatty acids labeled with iodine radioisotopes as tracers. In a study in rats, for example, Carter and colleagues (1990) evaluated alcohol’s effects on fatty acid uptake in the heart, liver, and small intestine. The researchers found that with increasing amounts of alcohol, fatty acid uptake by all three organs progressively decreased in a pattern related to the dose of alcohol received. This result suggests that chronic alcohol consumption may interfere with the heart muscle’s energy production, leading to a deterioration of the heart’s pumping capacity and, eventually, cardiomyopathy.

Kaiser and Feinendegen (1987), using another iodine-labeled fatty acid, showed that both acute and chronic alcohol consumption in humans were associated with altered myocardial fatty acid metabolism. Moreover, these changes were reversible after several weeks of abstinence. Studies such as these demonstrate that it is possible to evaluate alterations of myocardial fatty acid metabolism in humans noninvasively, using single-photon imaging approaches. One limitation of these techniques, however, is that they do not allow researchers to quantify precisely the metabolism of fatty acids in the heart, in part because the tracers used are not normal, physiological substances. Nevertheless, these approaches are useful for semiquantitative evaluations.

### Positron Emission Tomography

**Figure 2:**
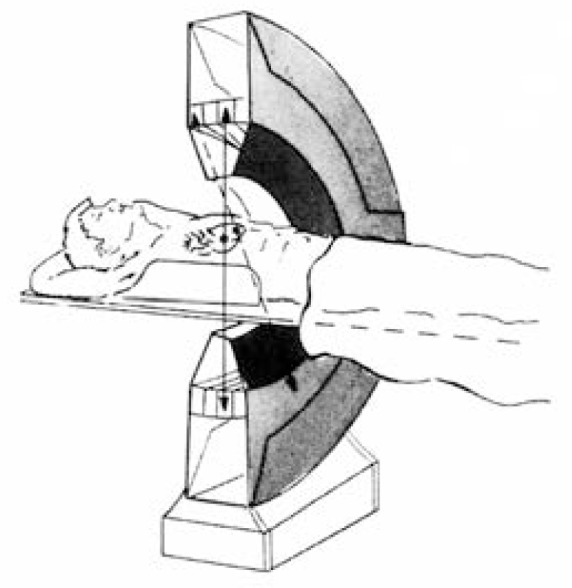
Schematic diagram of a positron emission tomography instrument. Radiolabeled substances injected into the patient’s body (indicated by asterisks in the heart) emit a certain type of radiation that can be detected by a complex array of detectors surrounding the patient. SOURCE: Reproduced with permission from Bergman 1990.

PET is a powerful nuclear medicine technique that allows absolute quantitation of myocardial blood flow and metabolism (Bergmann 1990). This technique (figure 2) also relies on radiolabeled substances; however, the radioisotopes used emit a different kind of radiation from those used for single-photon imaging techniques. The most commonly used radioisotopes are variants of oxygen, nitrogen, carbon, and fluorine, which can be incorporated into a wide variety of compounds of physiological interest, including fatty acids, sugars, and water. These radiolabeled compounds can then be used to study fatty acid or glucose metabolism as well as blood flow in the heart muscle. The radioisotopes also can be incorporated into medications and other substances to study the metabolism of these compounds. This flexibility makes PET an extremely attractive approach for investigating disorders and their possible treatments in intact, living organisms. The technique increasingly is being used to study the functions of the brain and the heart, among other organs.

### PET Studies of Myocardial Metabolism and Alcohol’s Effects

Because fatty acids play a central role in myocardial metabolism, numerous investigators have used a radiolabeled variant of one of the most abundant fatty acids found in human blood, palmitate (Bergmann 1990; Lerch 1992). Several studies assessed normal and abnormal myocardial fatty acid metabolism using this palmitate variant. For example, Lerch and colleagues (1992) demonstrated that PET could identify the decreased uptake and use of fatty acids during coronary artery blockage as well as the restoration of the heart’s metabolic function when the blood flow normalized after treatment with blood clot-dissolving drugs. Thus, PET is suitable for studying both the impairment and the recovery of organ function.

Geltman and colleagues (1983) used PET with radiolabeled palmitate as a tracer in patients with cardiomyopathy, which, in some patients, likely was attributable to chronic alcohol consumption. In all patients, the tracer accumulated unevenly (i.e., exhibited marked spatial heterogeneity) throughout the myocardium, indicating that myocardial metabolism was reduced in some regions of the heart muscle. This spatial heterogeneity may have been caused by the nonfunctional, fibrous tissue that replaces normal heart muscle late in the development of cardiomyopathy.

**Figure 3:**
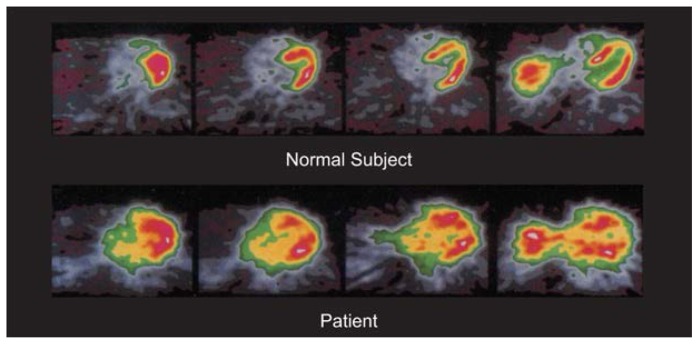
Positron emission tomography images of cross-sections through the heart of a normal subject (top) and of a patient with presumed alcohol-induced heart muscle disease (i.e., cardiomyopathy) (bottom) using a radiolabeled fatty acid, palmitate. The yellow and red colors in the normal subject represent the heart’s metabolism of radiolabeled palmitate. In contrast, the myocardium (i.e., heart muscle) of the patient with alcohol-induced cardio-myopathy is enlarged and does not process palmitate normally, suggesting defective metabolism. (The bright signals to the left of the hearts in the rightmost images represent the top of the liver.)

Recently, Bergmann and colleagues (in press) developed a mathematical model that permits quantitation of myocardial fatty acid metabolism based on PET imaging. This model also allows clinicians to assess the changes in myocardial fatty acid metabolism associated with a variety of diseases—including alcohol-induced heart muscle disease—in more detail. For example, PET could identify and quantify impaired metabolism in children with cardio-myopathy resulting from inherited defects in enzymes necessary for the metabolism of fatty acids (Bergmann et al. 1995*a*). The same technique also detected impaired uptake and metabolism of fatty acids from the blood in some patients with alcohol-induced heart muscle disease (figure 3) (Bergmann 1995*b*). The confirmation of these observations in an expanded number of subjects would allow researchers to determine whether abnormalities in fatty acid metabolism predict changes in the heart’s contraction and pumping ability. Similar studies could assess the correlation between recovery of the heart’s ability to effectively metabolize fatty acids (and potentially other substances) and the reversal of alcohol-induced heart muscle disease following abstinence or other therapeutic treatments. In addition, these findings, although still preliminary, suggest that PET eventually may allow researchers and clinicians to distinguish between different kinds of cardiomyopathy.

### Magnetic Resonance Imaging

MRI is another imaging approach that can help to noninvasively assess the metabolism of organs. Although used primarily to define the structure of organs such as the heart, as described previously, recent advances in MRI technology extend its usefulness to the study of organ function on the biochemical level. For example, MRI analyses using a specific variant of phosphorus have enabled researchers to study phosphorus-containing chemical compounds that play important metabolic roles in the body, such as adenosine triphosphate (ATP). ATP provides the energy for many chemical reactions in the body and is crucial for every cell’s metabolism. Meyerhoff and colleagues (1989) performed MRI studies using this phosphorus variant in patients with alcohol-induced liver disease. The study found that these patients had diminished ATP levels in the liver, suggesting reduced energy production or storage in this organ. Similar analyses also could be used to assess alcohol’s effects on myocardial metabolism.

Other MRI studies make use of a certain variant of carbon that can be detected by MRI and which can be incorporated into a variety of physiological substances, such as fatty acids or sugars. By administering physiological compounds containing this carbon variant, researchers in the future should be able to assess alcohol-induced metabolic disturbances in the heart and other organs.

## Conclusions

This overview has presented several imaging procedures that permit insight into both normal and pathological tissue structure and metabolism. Although these techniques have not been applied extensively to studying the effects of alcohol on blood flow or myocardial metabolism, they are readily amenable to such studies and are likely to be applied in the future. Use of these approaches should permit a greater understanding of the effects of alcohol on the heart and of the mechanisms underlying alcohol-induced heart muscle disease. Furthermore, these techniques eventually should allow the objective evaluation of therapeutic strategies for alcohol-related heart disease.

## Figures and Tables

**Table 1:** Imaging Modalities To Assess Heart Function

Modality	Advantages	Limitations
Echocardiography	Widely available Allows assessment of heart function during measurement	Cannot assess metabolism
Single-Photon Emission Imaging	Widely available Allows easy qualitative measurements	Uses radioactive tracers Requires use of nonphysiological substances as tracers Allows no quantitative measurements
Positron Emission Tomography	Allows quantitative measurements Uses physiological substrates as tracers	Uses radioactive tracers Limited availability Costly
Magnetic Resonance Imaging	Exquisite anatomic detail Becoming widely available Uses no radioactive tracers Ability to assess function, blood supply, and possibly metabolism in a single study	Limited sensitivity for measuring metabolism Costly

## Footnotes

1 One standard drink is defined as 12 fluid ounces of beer, 5 fluid ounces of wine, or 1.5 fluid ounces of distilled spirits.

2 The coronary arteries supply the heart muscle with blood.

3 Lipoproteins (e.g., cholesterol) are molecules found in the blood stream that are composed of a lipid component and a protein component; some lipoproteins have been associated with the development of artherosclerosis (“bad cholesterol”), while others may protect the body from artherosclerosis (“good cholesterol”).

4 Radiolabeled means that the substance contains a radioactive variant of a chemical element.

## References

[b1-arhw-19-4-287] Balkau B, Eschwege E, Ducimetiere P, Richard JL, Warnet JM (1991). The high risk of death by alcohol related diseases in subjects diagnosed as diabetic and impaired glucose tolerant: The Paris Prospective Study after 15 years of follow-up. Journal of Clinical Epidemiology.

[b2-arhw-19-4-287] Balkau B, Eschwege E, Fontbonne A, Claude JR, Warnet JM (1992). Cardiovascular and alcohol-related deaths in abnormal glucose tolerent and diabetic subjects. Diabetologia.

[b3-arhw-19-4-287] Bergmann SR, Gerson M (1990). Positron emission tomography of the heart. Cardiac Nuclear Medicine.

[b4-arhw-19-4-287] Bergmann SR, Herrero P, Hartman JJ, Rubin PJ, Kelly DP (1995a). Quantitative assessment of myocardial fatty acid metabolism in pediatric patients with inherited cardiomyopathy. (Abstract). Circulation.

[b5-arhw-19-4-287] Bergmann SR, Rubin PJ, Hartman JJ, Herrero P (1995b). Detection of abnormalities in fatty acid metabolism in patients with cardiomyopathy using PET. (Abstract). Journal of Nuclear Medicine.

[b6-arhw-19-4-287] Bergmann SR, Weinheimer CJ, Markham J, Herrero P Noninvasive quantitation of myocardial fatty acid metabolism using positron emission tomography. Journal of Nuclear Medicine.

[b7-arhw-19-4-287] Bing RJ (1982). Effect of alcohol on the heart and cardiac metabolism. Federation Proceedings.

[b8-arhw-19-4-287] Carter EA, Barli-Kovach M, Elmaleh D, Livni E, Strauss HW (1990). Acute alcohol ingestion reduces fatty acid extraction of the heart, liver, and small intestine. Alcoholism: Clinical and Experimental Research.

[b9-arhw-19-4-287] Geltman EM, Smith JL, Beecher D, Ludbrook PA, Ter-Pogossian MM, Sobel BE (1983). Altered regional myocardial metabolism in congestive cardiomyopathy detected by positron tomography. American Journal of Medicine.

[b10-arhw-19-4-287] Kaiser KP, Feinendegen LE (1987). Planar scintigraphy versus PET in measuring fatty acid metabolism of the heart. Hertz.

[b11-arhw-19-4-287] Lange LG, Bergmann SR, Sobel BE (1981). Identification of fatty acid ethyl esters as products of rabbit myocardial ethanol metabolism. Journal of Biological Chemistry.

[b12-arhw-19-4-287] Lerch RA, Bergmann SR, Sobel BE, Bergmann SR, Sobel BE (1992). Delineation of myocardial fatty acid metabolism with positron emission tomography. Positron Emission Tomography of the Heart.

[b13-arhw-19-4-287] Lieber CS (1995). Medical disorders of alcoholism. New England Journal of Medicine.

[b14-arhw-19-4-287] Meyerhoff DJ, Boska MD, Thomas A, Weiner MW (1989). Quantitative image-guided ^31^P magnetic resonance spectroscopy of alcoholic liver disease. Radiology.

[b15-arhw-19-4-287] Preedy VR, Richardson PJ (1994). Ethanol-induced cardiovascular disease. British Medical Bulletin.

[b16-arhw-19-4-287] Rubin E (1993). The chemical pathogenesis of alcohol-induced tissue injury. Alcohol Health & Research World.

[b17-arhw-19-4-287] Rubin E, Doria J (1990). Alcoholic cardiomyopathy: Clinical, pathological, and experimental aspects. Alcohol Health & Research World.

[b18-arhw-19-4-287] Urbano-Marquez A, Estruch R, Navarro-Lopez F, Grau JM, Mont L, Rubin E (1989). The effects of alcoholism on skeletal and cardiac muscle. New England Journal of Medicine.

[b19-arhw-19-4-287] Urbano-Marquez A, Estruch R, Fernandezsola J, Nicolas JM, Pare JC, Rubin E (1995). The greater risk of alcoholic cardiomyopathy and myopathy in women compared with men. Journal of the American Medical Association.

